# Neonatal Immune Incompatibilities between Newborn and Mother

**DOI:** 10.3390/jcm9051470

**Published:** 2020-05-14

**Authors:** Borros Arneth

**Affiliations:** 1 Institute for Laboratory Medicine, Pathobiochemistry and Molecular Diagnostics, Hospital of the Universities of Giessen and Marburg, Justus Liebig University, 35339 Giessen, Germany; borros.arneth@klinchemie.med.uni-giessen.de; 2 Institute for Laboratory Medicine, Pathobiochemistry and Molecular Diagnostics, Hospital of the Universities of Giessen and Marburg, Philipps University Marburg, Baldingerstraße 1, 35033 Marburg, Germany

**Keywords:** neonatal alloimmune thrombocytopenia (NAIT), neonatal alloimmune neutropenia (NAIN), morbus hemolyticus neonatorum

## Abstract

**Background**: Incompatibilities between the mother and unborn baby can cause complications that must be identified early to initiate the appropriate treatment. For example, neonatal alloimmune thrombocytopenia (NAIT), neonatal alloimmune neutropenia (NAIN), and morbus hemolyticus neonatorum affect children worldwide. **Aim**: This literature review aims to depict the similarities and differences between these three disorders from a clinical and mechanistic point of view. **Material and Methods**: The current literature review entailed conducting a systematic search to locate articles on the three conditions. Different electronic databases, including PsycINFO, PubMed, Web of Science, and CINAHL, were searched using the search terms “neonatal alloimmune thrombocytopenia”, “neonatal alloimmune neutropenia”, “morbus hemolyticus neonatorum”, “NAIT”, “FNAIT”, “fetal”, “NAIN”, and “hemolytic disease of the newborn”. **Results**: This review shows that these three diseases are caused by incompatibilities between the maternal and fetal immune systems. Furthermore, these conditions can lead to severe complications that hinder fetal development and cause death if not well managed. Discussion: The current literature review shows that NAIT, NAIN, and morbus hemolyticus neonatorum are rare conditions that occur when the mother produces antibodies against the fetal immune system. Thus, there is a need for the early detection of these conditions to initiate appropriate treatment before the child experiences adverse effects. **Conclusion**: The development of NAIT, NAIN, and morbus hemolyticus neonatorum is linked to the production of antibodies against the fetal immune system and fetal antigens. Further studies are required to determine potential interventions to reduce the risk of developing these three conditions.

## 1. Introduction

The focus of this literature review is antibody-mediated diseases that arise from incompatibilities between the mother and the unborn child. Maternal antibodies produced in the blood cross the placenta to the baby and cause problems in the fetal blood such as thrombopenia, neutropenia, and/or the lysis of erythrocytes. Neonatal alloimmune thrombocytopenia (NAIT) is a disorder linked to maternal antibodies and immune incompatibility between the unborn baby and the mother [1]. NAIT arises when maternal antibodies act against fetal platelet alloantigens [2,3]. Although most NAIT cases tend to be mild, this disorder can cause mortality and morbidity in newborns if not detected and managed in a timely and appropriate manner [2,3,4]. Another disorder that has been associated with an incompatibility between the unborn baby and the maternal immune system is neonatal alloimmune neutropenia (NAIN) [5,6,7,8], which has been associated with antagonism involving the neutrophils [6,7,8]. Recent investigations have reported that approximately 0.35–1.1% of NAIN cases are characterized by granulocyte-specific antibodies [7]. Finally, newborns can develop morbus hemolyticus neonatorum as a result of immune system incompatibility regarding the erythrocytes (blood type). [9,10,11]. The aim of this systematic review is to compare these three conditions and to depict the similarities and differences between these three disorders from a clinical and mechanistic point of view. So far, there is no review comparing all three conditions.

## 2. Methodology

**Figure 1:**
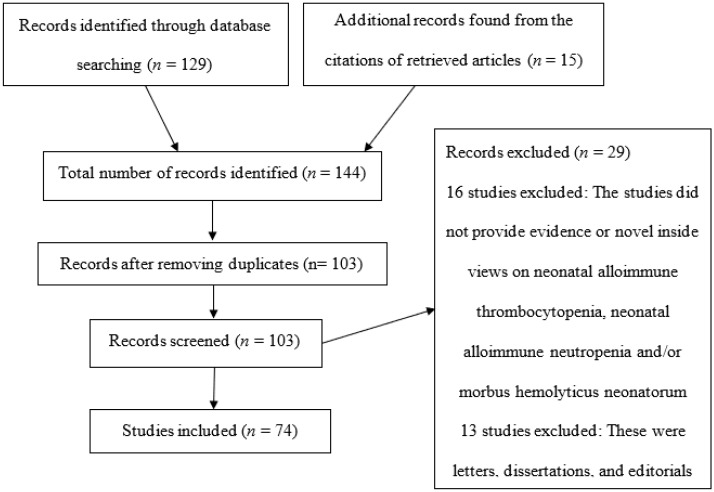
PRISMA flow diagram.

The process entailed analyzing the results of investigations related to the topic of interest published in PsycINFO, PubMed, Web of Science, and CINAHL. The search terms and phrases were “neonatal alloimmune thrombocytopenia”, “neonatal alloimmune neutropenia”, “morbus hemolyticus neonatorum", “NAIT", “FNAIT”, “fetal”, “NAIN”, and “hemolytic disease of the newborn”, and Boolean operators (AND/OR) were used to combine search terms to identify additional sources for the systematic review. The search was limited to articles published in the four electronic databases between 2009 and 2019. The abstracts of the available articles were carefully reviewed to determine their quality and appropriateness (Figure 1). The initial search yielded approximately 301 articles. Other parameters, including the study type, publication year, text options, search field tags, and language, were used to limit the search. When these parameters were applied, 129 articles were obtained from the database searches, and 15 were identified by cross-referencing. The articles were carefully examined in the different stages shown in Figure 1 to determine their suitability for this review.

At the end of the search and review process, the final list of articles consisted of prospective clinical trials, experimental studies, and clinical reviews. A total of 74 studies met the inclusion criteria and were considered for review. These articles provide vital insights into the development and progression of NAIT, NAIN, and morbus hemolyticus neonatorum. Table 1 shows a summary of the 23 most important papers that were selected and reviewed in this paper. 

## 3. Results

From the data collected, it is evident that incompatibility between the fetal and maternal immune systems can cause severe complications that hinder normal development and even lead to death [8,9]. Live-born affected children may experience developmental challenges and other complications that may adversely affect their chance of survival [10,11,29]. Consequently, studies are underway to ascertain the pathogenesis of these disorders and to identify management strategies. Furthermore, this review revealed that NAIT, NAIN, and morbus hemolyticus neonatorum are similar in terms of development, as they involve maternal antibodies against antigens on fetal red blood cells. A clear understanding of these disorders, however, can be more readily attained when they are individually analyzed in terms of mechanism and relevant molecules.

### 3.1. Neonatal Alloimmune Thrombocytopenia (NAIT) and Fetal Neonatal Alloimmune Thrombocytopenia (FNAIT)

NAIT and FNAIT are the most common reason for intracranial hemorrhage in full-term newborns [3]. Research shows that NAIT is also the leading cause of thrombocytopenia in neonates and fetuses [3,4]. Infants with severe NAIT show a wide range of symptoms, including low platelet count, florid petechial bleeding, and purpura [5]. Thrombocytopenia can occur in the absence of intravascular coagulation challenges, bacterial infection, viral infection, and other congenital disorders. Evidence from prospective and longitudinal studies has revealed that the severity of thrombocytopenia in infants at risk of developing NAIT may vary among cases [2,6]. However, it has been reported that NAIT is more common among children born to mothers with blood group A than among those born to mothers with blood group O [1]. However, further investigations are required to examine the risk of developing NAIT and its underlying pathogenesis.

There is consensus among researchers that NAIT is an immunological condition that occurs when the mother produces antibody-linked and antibody-mediated responses against the platelet-specific antigens in the fetal blood circulatory system [12]. NAIT develops when the mother lacks the specific antigen against which the immunological attack is directed. The risk of developing NAIT varies among ethnic groups. In the Caucasian community, the most immunodominant antigen is HPA-1a, which is responsible for approximately 85% of NAIT cases. Furthermore, 10% of NAIT cases have been linked to HPA-5b [1]. Irrespective of the actual cause, individuals who develop NAIT commonly experience intracranial hemorrhage, a condition that may lead to lifelong disability or death if not well managed. Intracranial hemorrhage may start at the end of the second trimester [30,31]. Without proper screening, intracranial hemorrhage may not be detected until the child is born. Consequently, poor antenatal care and management during pregnancy are considered risk factors that may increase the severity of NAIT.

In the last decade, there have been attempts to identify the antigens that are potentially associated with the pathogenesis of NAIT [1,31,32]. The antigens that cause NAIT are normally found on platelet membrane glycoproteins (GPs), such as the von Willebrand factor receptor, αIIb/β3 integrin, fibrinogen receptor, and a glycosylphosphatidylinositol (GPI)-anchored protein [1,32]. GPs usually interact with extracellular matrix proteins and coagulation factors in the cellular environment. In the long run, these interactions facilitate hemostasis [32,33]. Recent investigations have revealed that single amino acid substitutions in GPs can lead to maternal immunization during different stages of pregnancy [13,34,35]; these changes eventually lead to NIAT and affect normal development. The commonly identified GPs include CD109, GPIIIa, GPIIb, GPIbα, GPIa, and GPIbβ [1].

One common antigen class related to NAIT development that has been extensively evaluated in previous studies is the human platelet-specific antigen (HPA) class [36,37,38]. HPAs are platelet GP polymorphisms that can cause the production of maternal alloantibodies that attack fetal antigens [38,39,40]. Previous studies have shown that HPA-1a, which results from a proline/leucine substitution in the plexin–semaphorin–integrin domain of GPIIb/IIIa, can increase the risk of NAIT [41,42]. Animal model studies have revealed that fetal–maternal incompatibility for HPA-1a is the most common cause of NAIT among African and Caucasian people [43,44,45]. Only 2% of women are HPA-1a negative, but this places them at risk of developing antibodies linked to HPA-1a specificity [1]. Most cases involving HPA-1a antibodies are associated with women who are positive for the class II histocompatibility antigen DRB3*0101 (DR52a) [14,46]. This correlation is further related to the fact that these women express a Leu33-containing GPIIIa peptide with a high affinity for DRB3*0101. However, further studies are required to characterize HPA-1a and analyze the possible mechanisms through which it increases the risk of NAIT.

Recent studies have shown that approximately 95% of confirmed NAIT cases among Caucasian patients are caused by maternal immunization against HPA-1, -2, -3, -5, and -15 [47,48]. However, other studies of cases negative for common HPAs have identified other mutations that encode rare HPAs [1,46,48]. Moreover, 20 of these mutations have been identified and used as the basis for explaining the development and risk of NAIT [1]. Some of the mutations that have been explored in previous studies and used to examine the immunogenic elements of NIAT include HPA-4b, HPA-6b, HPA-10b, HPA-13b, and HPA-21b [15]. While it is anticipated that additional low-frequency HPAs associated with NAIT may be identified in the future, maternal sensitization to these HPAs will account for a limited number of NAIT cases in different populations.

Researchers have also discovered that the risk and pathogenesis of NAIT are related to ABO antigens. Research has shown that some people with blood group A or B tend to have platelets that carry antigens that may be incompatible with maternal antigens [1,7], and these people have high levels of platelet A1 and B antigens [1]. These findings have raised the possibility that children with Type II high-expresser traits are at high risk of developing NAIT. However, further research is needed to determine the circumstances under which ABO antigens increase the risk of NAIT.

### 3.2. Modified anti-HPA-1 antibodies in Fetal Neonatal Alloimmune Thrombocytopenia (FNAIT)

Kapur et al. have demonstrated in 2014 that a prominent lack of IgG-fucosylation of anti-HPA-1a antibodies is present in sera of FNAIT patients [16]. This lack of fucosylation causes an enhancement of the antibody-mediated phagocytosis of platelets through increased binding of the antibodies on platelets to FcgRIIIa/b. Thereby the degree of anti-HPA-1a fucosylation positively correlates with the neonatal platelet counts in FNAIT and negatively correlates to the clinical disease severity.

Analogously Bakchoul et al. have demonstrated the importance of a deglycosylated monoclonal anti-HPA-1a antibody from a therapeutic point of view in a mouse model [17]. Deglycosylation of antibodies abrogates the Fc-related effector functions. Therefore an Fc inactive deglycosylated monoclonal anti-HPA-1a antibody could potentially serve as a therapeutic tool in FNAIT in the near future by competitively inhibiting the binding of maternal alloantibodies [17].

Furthermore, in FNAIT neonates, the antibody titer does NOT strictly correlate with the clinical disease severity as with intracerebral hemorrhages. Intracerebral hemorrhage, thereby, is the most feared complication of FNAIT neonates. Thus other factors are likely involved in this respect, such as the IgG-FC-glycosylation patterns and other factors, as discussed by Sachs and Santoso in 2017 [49]. In addition, the role of anti-endothelial antibodies as a cause of intracerebral hemorrhage in FNAIT has been discussed by Santoso et al. in 2016 [18]. 

### 3.3. Neonatal Alloimmune Neutropenia

In some cases, incapability between the maternal and fetal immune systems results in NAIN. Although this condition is rare, it can adversely affect fetal development and lead to death [7]. The main signs of NAIN include pneumonia, meningitis, sepsis, and omphalitis. Furthermore, NAIN can lead to skin infections. Interestingly, NIAN is linked to cases in which the mother develops alloantibodies against the neutrophil antigens inherited by the fetus from the father [8,9]. This condition may be more severe if the father is heterozygous for the antigen, which causes incompatibility between the mother and fetus [10,11,50]. Like other autoimmune disorders, NAIN has a complex pathogenesis and underlying mechanism of development [19,51,52]. Thus, there are continued research and clinical development efforts to gather additional information on NAIN [53,54,55]. There is consensus among researchers that NAIN usually occurs when maternal antibodies are sensitized to fetal neutrophils in the fetus [56,57,58,59]. Antibodies in the immunoglobulin G (IgG) class are transported across the placenta. Once these antibodies reach the fetus, they act on fetal neutrophils, causing cellular destruction [20,60,61]. Furthermore, these antibodies can inhibit granulocytopoiesis. In certain instances, NAIN is linked to isoantibodies such as HNA-2 and/or other HNA antibodies.

Researchers have identified different classes of human neutrophil antigen (HNA) associated with the development of NAIN [21,22,62]. Currently, a total of 11 HNAs grouped into five HNA classes, HNA-1, HNA-2, HNA-3, HNA-4, and HNA-5, have been identified in previous studies and linked to NAIN development. The HNA-1 group is usually carried through Fc gamma receptor IIIb (FcγRIIIb) [63,64].

In this class, four primary HNAs have been associated with the development of NAIN, including HNA-1a, b, c, and d. A combination of these HNAs has been observed in patients with NAIN.

The second class is the HNA-2 group that contains two variants, the HNA-2-positive and CD177 (HNA-2)-negative neutrophil groups [23,24,65,66].

The third category is the HNA-3 system, which consists of two antigens, HNA-3a and HNA-3b. In other cases, the development of NAIN is linked to the HNA-4 system, which consists of the HNA-4a and HNA-4b alleles. Finally, the HNA-5 system contains a single antigen, HNA-5a, that has also been implicated in NAIN development [67,68]. A review of previous studies shows that any HNA group can increase the risk of NAIN [69]. However, only a few cases of NAIN have been linked to the production of HNA-3a, HNA-3b, HNA-4b, and HNA-5a. Antibodies against these neutrophils can be detected through the leucoagglutination technique. In other studies, researchers used monoclonal antibody immobilization of granulocyte antigens (MAIGA) and the granulocyte immunofluorescence test (GIFT) to identify maternal antibodies that potentially attack fetal HNAs and lead to the development of NAIN [7].

### 3.4. Morbus Hemolyticus Neonatorum

Morbus hemolyticus neonatorum is an alloimmune condition that occurs when maternal IgG attacks antigens on fetal red blood cells. Notably, IgG is one of the main antibodies produced by the mother [25,26], and it can target antigens in the fetal circulation in cases of complete incompatibility [25,26,27,28]. In such cases, IgG breaks down and destroys the antigens on fetal red blood cells through hemolysis. Over time, the fetus commonly develops anemia or reticulocytosis [30]. The severity of the disorder may vary among cases [27,28], and this disorder can lead to complications such as heart failure or even death. Morbus hemolyticus neonatorum, similar to NAIT and NIAN, is a disorder associated with immune system incompatibility or impairment that affects immune tolerance during pregnancy [26,28]. Thus, these conditions can adversely impact fetal development and lead to severe complications or even death [26].

Research evidence shows that morbus hemolyticus neonatorum is primarily caused by Rhesus incompatibility between the mother and child [25,26,70,71]. In some cases, however, this disorder has been associated with both Rhesus incompatibility and fetal alloimmune thrombocytopenia [25,26,72]. In such instances, the child may show low fetal hemoglobin and platelet levels. It is imperative that treatment is initiated early to ensure the survival of the child; treatment may entail the transfusion of packed red cells and platelets through the umbilical vein.

### 3.5. Therapeutic Strategies

NAIT, NAIN, and morbus hemolyticus neonatorum can adversely affect the wellbeing of infants. Thus, research is underway to explore interventions for disease management [7]. For NAIT, the standard intervention is intravenous human immunoglobulin (IVIG) administration, which aims to increase fetal platelet count [46]. However, the actual mechanism through which IVIG alleviates NAIT is not yet fully understood. NAIN, on the other hand, is managed by prophylactic treatment and granulocyte-colony stimulating factor (G-CSF) [56,59]. 

For NAIT / FNAIT patients, IVIg is only administered in second and subsequent pregnancies, so far we do not have a reliable test to predict clinical disease severity of NAIT in first pregnancies. 

The administration of G-CSF can increase the neutrophil population, thus improving the survival rate of affected children [60]. Finally, morbus hemolyticus neonatorum can be managed through increased fluid intake, light therapy, and IVIg administration to protect the child’s red blood cells from destruction [71,72,73]. In other cases, researchers have suggested that exchange transfusion can be used to manage morbus hemolyticus neonatorum [74]. However, further investigations are needed to gather additional information on the efficacy of various interventions.

## 4. Discussion

NAIT, NAIN, and morbus hemolyticus are conditions that may affect normal fetal development [1,15,26,73]. The main similarity among these conditions is that they are caused by incompatibilities between the mother and the unborn child (Table 2). NAIT occurs due to the passive transfusion of maternal antibodies that eventually target platelet antigens [74], and NAIN arises when maternal anti-neutrophil antibodies become sensitized to fetal neutrophils, which leads to the destruction of these cells. Cases of both conditions are rare in part because only a few HLA antibodies can cause NAIT. The current review reveals that NAIT is considered the platelet analog of the Rhesus incompatibility that causes morbus hemolyticus neonatorum.

Thus, there is a need to carefully screen the fetus in the early stages of the pregnancy to determine the possible risks of NAIT, NAIN, and morbus hemolyticus.

## 5. Conclusions

Different conditions occur during pregnancy and may affect the normal development of the fetus. In some cases, the antibody-mediated diseases arising from incompatibilities between the mother and the unborn child may lead to complications during and after pregnancy. NAIT, NAIN, and morbus hemolyticus are examples of the diseases that occur due to maternal and fetal incompatibilities. While the disorders are considered to be antibody-mediated diseases, the actual causes differ in each case. Thus, expectant mothers should be carefully screened to determine the presence of antibodies that may lead to the occurrence of NAIT, NAIN, and morbus hemolyticus. Further studies are also required to identify new interventions that can help reduce the risk of these three conditions and prevent adverse effects.

## Figures and Tables

**Table 1:** Summary of the 23 most important studies.

Author	Design	Findings
Peterson et al. (2013) [1]	Review	The authors opined that different kinds of the first human platelet antigen (HPA) have been linked to the pathogenesis of NAIT. The identification of the HPAs that increase the risk of developing the disease provides an avenue through which the disease can be diagnosed and managed.
Ahlen et al. (2012) [2]	Correlational study	The researchers reported that there was a significant link between the risk of NAIT due to anti-HPA-1a antibodies and the maternal blood type. The risk of NAIT was high among pregnant women with blood type A.
Arinsburg, Shaz, Westhoff, and Cushing (2012) [3]	Review	The study showed that NAIT was a major cause of intracranial hemorrhage and severe cases of thrombocytopenia. The disorder can be detected through the use of the HPA-specific antibodies and platelet genotyping with the sequence-specific primer-polymerase chain reaction (PCR-SSP) approach.
Bakchoul et al. (2011) [4]	Retrospective cohort analysis and a NOD/SCID mouse model of alloimmune thrombocytopenia	Low-avidity HPA-1a antibodies are present in a significant number of NAIT cases and, although they can escape detection by standard serology, they harbor the capability of PLT destruction in mice.
Porcelijn and de Haas (2018) [8]	Review	A review of prospective screening studies showed that granulocyte-specific antibodies that caused NAIN were present in approximately 0.35–1.1% of the maternal samples. Furthermore, the researchers stated that the incidence of the disease was below 0.1%.
Tomicic et al. (2014) [10]	Prospective study	The researchers detected anti-HNA antibodies in approximately 54% of the samples that were proven to be serologically positive for alloimmune neonatal neutropenia (ANN) between 1998 and 2008.
Bussel and Sola-Visner (2009) [12]	Review	The researchers stated that if a mother gives birth to a child with alloimmune thrombocytopenia, there are high chances that the next child will also develop severe NAIT.
Espinoza, Caradeux, Norwitz, and Illanes (2013) [13]	Review	FNAIT is a rare fetal complication that develops when a woman is alloimmunized against the platelet antigens in the fetus.
Tiller et al. (2016) [14]	Prospective observational study	The authors found that there was an increase in the neonatal platelet count in HPA-1a immunized women during their subsequent pregnancies.
Peterson et al. (2012a) [15]	Observational study	The study showed that HPA-21bw and HPA-4b were common triggers of NAIT among Caucasian women. The production of maternal antibodies against these antigens can lead to the development of NAIT.
Kapur et al. (2014) [16]	Human patient study (*n* = 48)	The study showed markedly decreased levels of the fucosylation of the anti-HPA-1a specific IgG1 in FNAIT patients. Antibodies with a low amount of Fc fucose showed enhanced phagocytosis of platelets. A positive correlation of anti-HPA-1fucosylation with neonatal platelet counts was found as well as a negative correlation of anti-HPA-1fucosylation with the clinical disease severity.
Bakchoul et al. (2013) [17]	Mice study and human cell study	In FNAIT, platelet destruction is mediated via the Fc part of the anti-HPA alloantibodies. Deglycosylation of antibodies abrogates the Fc-related effector functions. Deglycosylation of SZ21 abrogates Fc-effector functions without interfering with placental transport or the ability to block anti–HPA-1a binding. A therapeutical use of such an antibody might be possible.
Santoso et al. (2016) [18]	Human patient study (*n* = 36) Antibodies from mothers with ICH-positive FNAIT and with ICH-negative FNAIT were investigated and compared	The authors found a stronger binding of +ICH antibodies to endothelial cell-derived αvβ3. By absorption experiments, anti-HPA-1a antibodies with anti-αvβ3 specificity were found in the ICH positive, but not in the ICH negative cohort. Only the anti-αvβ3 subtype, but not the anti-β3 subtype was found to be able to induce epithelial cell apoptosis of HPA-1a positive epithelial cells. The maternal anti-HPA-1a subtype seems to determine the risk for ICH development of the child.
Winkelhorst, Oepkes, and Lopriore (2017) [19]	Review	The researchers stated that the optimal intervention for the management of FNIAT was noninvasive treatment involving the weekly intravenous administration of immunoglobulin. A dose of 0.5 or 1.0 g/kg should be given to prevent aggravation of the condition.
Chaudhuri et al. (2012) [20]	Randomized controlled trial	Chaudhuri et al. (2012) concluded that the mortality factor in the granulocyte colony-stimulating factor (GCF) group was significantly lower than the rate in the control group (10% vs. 35%).
Atkas et al. (2015) [21]	Randomized case-controlled study	The study revealed that treatment with recombinant human granulocyte colony-stimulating factor therapy resulted in rapid recovery from sepsis among neutropenic children.
Curtis et al. (2016) [22]	Case study	The sera analysis led to the detection of IgG antibodies in women with HNA-4b+ neutrophils.
Regan et al. (2019) [23]	Review	NAIT occurs when the immune system of the mother fails to recognize the baby’s HPAs inherited from the father. In such instances, the mother develops antibodies that can cross the placenta and attack the fetal HPAs.
Del Vecchio and Christensen (2012) [24]	Review	The researchers opined that the early onset of neutropenia in infants was linked to cases of severe sepsis, asphyxia, periventricular hemorrhage, and maternal hypertension.
Basu, Kaur, and Kaur (2012) [25]	Review	The scholars found out that hemolytic disease occurs as a result of Rhesus incompatibility between the mother and the fetus.
Arora et al. (2015) [26]	Case study	Morbus hemolyticus neonatorum develops due to maternal alloimmunization, a process that adversely affects the development of the fetus.
Gowri et al. (2015) [27]	Retrospective study	Gowri et al. stated that Rhesus incompatibility could lead to a wide range of complications such as jaundice, neonatal anemia, and respiratory distress syndrome
De Haas et al. (2015) [28]	Review	The study showed that morbus hemolyticus neonatorum was caused by maternal alloimmunization against the fetal red blood cell antigens. The disorder could lead to anemia, icterus, and fetal death.

**Table 2:** Summary of the major findings.

Platelet-Specific Antigens Associated with NAIT	Neutrophil Antigens Associated with NAIN	Risk Factor for Morbus Hemolyticus Neonatorum
HPA-1a [1,2] HPA-2b [1] HPA-3a [3,4] HPA-3b [3,4] HPA-4b [5,6] HPA-5a [1] HPA-6b [8] HPA-10b [10] HPA-13b [30] HPA-15 [32] HPA-16 [15,32] HPA-21b [1]	HNA-1a, b, c, and d [7] HNA-2 positive and HNA-2-negative (CD177) [19,52] HNA-3a and HNA-3b [7] HNA-4a and HNA-4b [53,54,55] HNA-5a [54,56]	Rhesus incompatibility [67,68]
